# A signature motif mediating selective interactions of BCL11A with the NR2E/F subfamily of orphan nuclear receptors

**DOI:** 10.1093/nar/gkt761

**Published:** 2013-08-23

**Authors:** Chun Ming Chan, Joel Fulton, Cristina Montiel-Duarte, Hilary M. Collins, Neetu Bharti, Frances R. Wadelin, Paula M. Moran, Nigel P. Mongan, David M. Heery

**Affiliations:** ^1^Gene Regulation Group, Centre for Biomolecular Sciences, School of Pharmacy, University of Nottingham, Nottingham NG7 2RD, UK, ^2^School of Psychology, University of Nottingham, Nottingham NG7 2RD, UK and ^3^School of Veterinary Medicine and Science, University of Nottingham, Nottingham NG7 2RD, UK

## Abstract

Despite their physiological importance, selective interactions between nuclear receptors (NRs) and their cofactors are poorly understood. Here, we describe a novel signature motif (F/YSXXLXXL/Y) in the developmental regulator BCL11A that facilitates its selective interaction with members of the NR2E/F subfamily. Two copies of this motif (named here as RID1 and RID2) permit BCL11A to bind COUP-TFs (NR2F1;NR2F2;NR2F6) and Tailless/TLX (NR2E1), whereas RID1, but not RID2, binds PNR (NR2E3). We confirmed the existence of endogenous BCL11A/TLX complexes in mouse cortex tissue. No interactions of RID1 and RID2 with 20 other ligand-binding domains from different NR subtypes were observed. We show that RID1 and RID2 are required for BCL11A-mediated repression of endogenous γ-globin gene and the regulatory non-coding transcript Bgl3, and we identify COUP-TFII binding sites within the Bgl3 locus. In addition to their importance for BCL11A function, we show that F/YSXXLXXL/Y motifs are conserved in other NR cofactors. A single FSXXLXXL motif in the NR-binding SET domain protein NSD1 facilitates its interactions with the NR2E/F subfamily. However, the NSD1 motif incorporates features of both LXXLL and FSXXLXXL motifs, giving it a distinct NR-binding pattern in contrast to other cofactors. In summary, our results provide new insights into the selectivity of NR/cofactor complex formation.

## INTRODUCTION

Nuclear receptors (NRs) are structurally dynamic proteins that selectively recruit coactivators and corepressors to regulate gene expression. Docking of selected cofactors with the NR ligand-binding domains (LBDs) is mediated by LXXLL (1,2) and CoRNR box signature motifs (3–5). Reports in the literature have identified potentially several hundred cofactors that bind one or more of the 48 known human NRs (www.NURSA.org). While some cofactors can exhibit relatively promiscuous [e.g. p160s, RIP140 (1,2)] or highly selective [e.g. Moses (6)] NR binding preferences, surprisingly little is known regarding how such molecular selectivity is achieved.

We have investigated the molecular basis of interactions between the orphan receptor COUP-TFII and the Krüppel zinc finger protein BCL11A (also known as CTIP1 and Evi9) (7). BCL11A is a developmental regulator expressed in haematopoietic and neural tissues whose overexpression is associated with acute leukaemia and B-cell lymphoma (8,9). Inactivating mutations in the BCL11A gene are associated with sickle cell disease and β-thalassemias (10–12), and it was subsequently shown that BCL11A is a master regulator of fetal haemoglobin switching (13,14). This is due to its function as a corepressor through association with transcription factors such as COUP-TFII/NR2F2 (7), GATA1 and SOX6 (13), although the molecular basis of these interactions is not well understood. Moreover, BCL11A and its homologue BCL11B/CTIP2 may act in conjunction with other regulators such as BCL6 (9), HP1α (15), SIRT1 (16), SUV39H1 (17), FOG1 (13) or the NuRD complex (18,19).

The COUP-TF/NR2F subfamily of NRs has a wide range of functions in development, reproduction and homeostasis (20). Based on sequence homologies, they are closely related to the RXR/NR2B and HNF4/NR2A proteins, as well as the NR2E proteins TLX/NR2E1 and PNR/NR2E3 (20,21). COUP-TFII is widely expressed in mesenchymal tissues of the developing embryo and is essential for angiogenesis and organogenesis including retinal and neural development (22). BCL11A also shows high expression in the developing and adult brain (7,23,24). Previous work from Leid and colleagues revealed that BCL11A and its homologue BCL11B are both capable of direct binding to COUP-TFII and that these proteins are present in high-molecular-weight corepressor complexes in neuroblastoma cells and other cell types (18,25). To understand the molecular basis of BCL11A/COUP-TFII functional interactions in more detail, we therefore set out to characterize the sequences that facilitate the formation of this complex using protein–protein interaction mapping studies.

## MATERIALS AND METHODS

### Plasmid constructs

Mammalian cell expression plasmids pcDNA3.1 FLAG-BCL11A-XL, pcDNA3.1 HA-TLX and pcDNA3.1 HA-PNR-(89–410) were constructed by cloning modified PCR fragments into the pcDNA3.1 vector. The FLAG-BCL11A-XL L319A/Y656A/W659A, L318A and Y656A/W659A and GFP-BCL11A-XL L319A/Y656A/W659A constructs were generated by site-directed mutagenesis. Plasmids pCH110-lacZ (26), pRARβ2-Luc (27) and pTL1-COUP-TFII (28) were gifts from E. Kalkhoven, G.Folkers and M. Leid, respectively. Full-length cDNA for BCL11A-XL and a GFP-BCL11A-XL construct were provided by M.J.S. Dyer (U. Leicester).

For yeast two-hybrid expression, PCR fragments encoding the required sequences were subcloned in-frame into plasmids pBTM116mod and pASV3mod to generate LexA DNA binding domain (DBD) and VP16 acidic activation domain (AAD) fusion constructs, respectively. Construction of the LexA-SRC1-NID-(431–761) expression vector has been described previously (29). LexA–BCL11A-[(212–290), (212–295), (212–313), (212–329), (212–376), (283–290), (295–376), (313–376), (326–376), (283–329), (302–325), (306–225), (310–325), (594–707), (594–687), (594–670), (611–641), (651–707), (651–687) and (651–670)], LexA-NSD1-(790–818) and LexA-COUP-TFII-LBD-(144–414) were generated by PCR subcloning, or by ligation of phosphorylated annealed oligonucleotide pairs into the pBTM116mod vector. Site-directed mutagenesis was used to introduce substitution mutations into constructs LexA-BCL11A-(302–325), LexA-BCL11A-(310–325), LexA-BCL11A-(651–670) and LexA-NSD1-(790–818). These include mutations in RID1 (F315A; S316A; R317A; L319A; R320A; L322A), RID2 (V655A; Y656A; S657A; Q658A; W659A; L660A; G662A; Y663A) and NSD1 NR interaction domain (Y801A; K802A; F803A; S804A; L806A; L807A; M808A; M809A; L810A; K811A; D812A). The following AAD-fusion constructs were generated by subcloning PCR fragments into the pASVmod vector: VDR-LBD-(123–426), DAX-1-(1–140), SHP-(106–461), Rev-erbβ-LBD-(187–408), RORα-LBD-(106–469), RORβ-LBD-(98–459), HNF4γ-LBD-(88–408), TR2-LBD-(195–467), TR4-LBD-(218–530), TLX-LBD-(100–385), PNR-LBD-(131–410), COUP-TFI-LBD-(151–467), COUP-TFII-LBD-(144–414), EAR2-LBD-(121–399), ERRα-LBD-(169–423), Nur77-(1–599), NURR1-(1–598), LRH-1-LBD-(133–495) and GCNF-LBD-(138–475). AAD-COUP-TFII-LBD C-terminal deletions Δ12-(144–392), Δ11-(144–380) and Δ10-(144–359) or substitution mutations (F221A; V224A; R228A; I238A; V242A; R246A) were generated by PCR and site-directed mutagenesis. For PCR templates, NR cDNA vectors were purchased from Open Biosystems. Constructs AAD-[ERα-LBD-(282–595), AR-LBD-(626–919), RARα-LBD-(200–462), RXRα-LBD-(200–462), TRβ-LBD-(169–456) and PPARγ-LBD-(173–475)] were described previously (29). NSD1 template cDNA was a gift from F. Cammas and R. Losson.

GST-COUP-TFI-(151–467), GST-COUP-TFII-(144–414), GST-PNR-(89–410) and GST-TLX-(100–385) were constructed by PCR subcloning into pGEX-DMH, a modified version of pGEX-2TK vector (30). The construct pGEX-2TK-GST-PPARγ-LBD-(173–475) (31) was a gift from E. Kalkhoven.

All constructs generated by PCR were sequenced to confirm their validity. The expression of fusion proteins in yeast was monitored by western blotting (See Supplementary Figures S1 and S2) using antibodies recognizing VP16 AAD (Santa Cruz SC7545) or LexA DBD (Millipore 06-719) as described previously (1).

### Yeast two-hybrid interaction assays

*Saccharomyces cerevisiae* L40 (trp1, leu2, his3, ade2, LYS2::(lexAop)_4×_-HIS3, URA3::(LexAop)_8×_-LacZ) was co-transformed with LexA-fusion and AAD-NR-fusion expression vectors using the lithium acetate method as described (1). Single transformants containing the desired plasmids were selected on appropriate media and grown to late log phase in 15 ml of selective medium (yeast nitrogen base containing 2% w/v glucose and appropriate supplements) in the presence of 10^−6^ M cognate ligand or vehicle. Preparation of cell-free extracts was by the glass bead method, and β-galactosidase assays were performed as described (1). Reporter β-galactosidase activities in the presence or absence of ligand were determined for three individual transformants for each condition, in replicated experiments as stated. Ligands for RARα (all-trans retinoic acid), RXRα (9-cis retinoic acid), ERα (17-β-estradiol), AR (mibolerone), PPARγ (roziglitazone), TRβ (Triiodothyronine) and VDR (cholecalciferol) were purchased from Sigma.

### GST pull-down assays

GST fusion proteins were expressed in *Escherichia coli* BL21 using isopropyl-β-d-thiogalactopyranoside induction, and purified on glutathione-Sepharose beads (Amersham Biosciences). The pcDNA3.1 BCL11A-XL and associated mutant expression vectors were *in vitro* transcribed/translated in the presence of [^35^S]-methionine in reticulocyte lysate (Promega) according to the manufacturer’s instructions. Equalised amounts of GST proteins were incubated with ^35^S-radiolabelled protein in NETN buffer (20 mM Tris, pH 8.0, 100 mM NaCl, 1 mM EDTA, 0.5% NP-40) containing 1× complete protease inhibitors (Roche Molecular Biochemicals) in the presence or absence of 10^−6^ M cognate ligand as described previously (32). Samples were washed three times, and bound proteins were separated by SDS-PAGE. Radiolabelled proteins in dried gels were visualized by autoradiography.

### Cell culture, transient transfections, reporter assays

U2OS and HEK293 cells were cultured in DMEM supplemented with 10% fetal bovine serum (FBS) and maintained as described previously (30). Twenty-four hours prior to transfection, cells were replated in phenol red-free DMEM supplemented with 5% dextran charcoal-stripped FBS. Transient transfections were performed using calcium phosphate co-precipitation. For reporter assays, transfected DNA included pCH110-lacZ internal control plasmid (500 ng/well), pRARβ2-Luc (100 ng) luciferase reporter plasmid and varying amounts of pTL1-COUP-TFII, pcDNA3.1 HA-TLX, pcDNA3.1-FLAG-BCL11A-XL or FLAG-BCL11A-XL L319A/Y656A/W659A as indicated. Empty pcDNA3.1 expression vector was used to standardize the amount of transfected DNA. After 16 h, fresh medium containing either 10^−^^6^ M all trans-retinoic acid ligand (ATRA) or vehicle was added. After a further 24 h, cells were harvested and cell-free extracts were prepared and assayed for luciferase activity using the Dual light® Luciferase Assay System (Applied Biosystems) and normalised to β-galactosidase activities. Reporter assays were performed in triplicate.

### Immunoprecipitation and western blots

For co-immunoprecipitation of recombinant proteins*,* HEK293 cells were co-transfected with pTL1-COUP-TFII, pcDNA3.1 HA-TLX or pcDNA3.1 HA-PNR-(89–410) in combination with pcDNA3.1 FLAG-BCL11A-XL or mutants. At 48 h post-transfection, the cells were washed in PBS, harvested and nuclear extracts prepared (2 mg) in 500 µl with Triton X-100 lysis buffer (50 mM Tris-HCl pH8, 150 mM NaCl, 1% Triton X-100, 1 mM PMSF, 1 mM DTT and 1× protease inhibitor cocktail). Lysates were equalised for protein content, pre-cleared by incubation with protein-G Plus Agarose (Santa-Cruz) and FLAG-tagged proteins were immunoprecipitated using 1 μg of anti-FLAGM2 (A2220, Sigma) and 20 μl Protein-G agarose overnight with gentle agitation. Agarose beads were then washed five times in ice cold PBS and precipitated proteins resolved by SDS-PAGE and detected by Western blotting using anti-BCL11A (14B5, Santa Cruz), anti-COUP-TFII (H7147, Abcam), anti-TLX (S-23, Santa Cruz or anti-HA (F-7, Santa Cruz) as appropriate.

To assess co-immunoprecipitation of endogenous proteins, dissected brain regions from wild-type mice were snap frozen on dry ice and homogenized in Triton X-100 lysis buffer (50 mM Tris-HCl pH 8, 150 mM NaCl, 1% Triton X-100, 1 mM PMSF, 1 mM DTT, 1× protease inhibitors), sonicated and cleared by centrifugation. Equalised amounts of protein were immunoprecipitated with the appropriate antibody as described above. The precipitates were resolved by SDS-PAGE for Western blot analysis.

### Immunohistochemistry

A 3-month-old male C57BL/6 J mouse was anaesthetized and sacrificed by transcardial perfusion with 4% paraformaldehyde in PBS. The brain was removed and immersion fixed in the fixative for 24 h at 4°C. Coronal tissue sections were prepared after embedding in paraffin. Immunohistochemical staining was performed using a *Dako* Envision kit following manufacturer’s instructions, and incorporating a microwave antigen retrieval step [10 min in 10 mM sodium citrate (pH 6.0)]. Primary antibody α-BCL11A (14B5, Santa Cruz) was used at a dilution of 1:50 and detected using DAB (brown) staining. Sections were counterstained with haematoxylin (blue). Tissue extractions were performed in accordance with local and national rules under the Animals (Scientific Procedures) Act UK 1986.

### Electrophoretic mobility shift assay

Forward and reverse strands of nucleic acid probes were subjected to 5′-hydroxyl end labelling with [γ^32^P]-ATP using T4 polynucleotide kinase. The labelled probes were annealed and purified on G-25 sephadex columns, before being adjusted to 100 000 cpm/μl for use in binding assays. Sequences of the probes used in this study are listed in Supplementary Figure S3A. Nuclear extracts (NE) were prepared from HEK293 cells transfected with pTL1-COUP-TFII, pcDNA3.1 HA-TLX, pcDNA3.1 FLAG-BCL11A-XL or pcDNA3.1 (Mock). Expression of the recombinant proteins were verified by western blots (Supplementary Figure S4B). Indicated amounts of NE were incubated with 1 µl nucleic acid probe, 1 μg poly(dI:dC)·(dI:dC) and 3 μg bovine serum albumin in electrophoretic mobility shift assay (EMSA) buffer (20 mM HEPES-KOH pH 7.9, 12% v/v glycerol, 50 mM KCl and 1 mM DTT) to a final volume of 10 µl. Binding mixes were incubated at room temperature for 20 min to allow DNA/protein complex formation. Where appropriate, specific antibodies for FLAG (A2220, Sigma) or COUP-TFII (H7147, Abcam) were added to binding mixes to validate the identity of protein/DNA complexes. Binding mixes were loaded onto 5% polyacrylamide gels and electrophoresed in 0.5× TBE prior to being visualized by autoradiography.

### Chromatin immunoprecipitation

Chromatin was prepared from DOHH2 or K562 cells grown in RPMI supplemented with foetal bovine serum. Approximately 1.5 × 10^7^ cells per condition were fixed with 1% formaldehyde for 8 min at room temperature and subsequently quenched with 0.125 M glycine. Cells were washed (×3) in ice-cold PBS, resuspended in cell lysis buffer (5 mM Tris-Cl (pH 8.0), 85 mM KCl, 0.5% NP-40) and incubated for 10 min on ice. Samples were then harvested at 1000 rpm for 10 min at 4°C and the supernatant aspirated. Chromatin was extracted from the nuclear pellet by resuspension in nuclei lysis buffer (50 mM Tris-Cl (pH 8.0), 10 mM EDTA, 1% SDS) supplemented with EDTA-free protease inhibitors (Roche) for 10 min on ice. Chromatin was sonicated using a Diagenode water bath sonicator under conditions optimized to give a DNA fragment length of 200–500 bp. DNA content was measured using a bioanalyser (Agilent) and fragment size verified by gel analysis.

Immunoprecipitation was performed with PureProteome magnetic beads (Millipore) as per manufacturer’s protocol. In all, 10–25 µg of chromatin was used for each IP which was incubated with 10 µg of specific antibody for COUP-TFII (Active motif, 61213) or BCL11A (Abcam, 19489) overnight at 4°C. The beads were washed (×3) for 10 min in 1× PBS-Tween and the immunoprecipitate was eluted in 60 µl elution buffer (0.2 M glycine pH 2.5). Samples were then neutralized with 1 M Tris (pH 8.5), reverse-crosslinked by incubating at 65°C overnight and treated with protease K. DNA was purified through Macherey-Nagel PCR purification kit according to manufacturer’s protocol. One microlitre of ChIP-enriched DNA was used in a 20 µl quantitative real-time polymerase chain reaction (qPCR) reaction as described previously (33). qPCR reactions were done in triplicate, and the samples were analysed on a 2% agarose gel to verify that PCR products were of the expected size. Primers were designed to amplify regions selected by bioinformatic analysis, to detect recruitment of factors at the human β-globin locus. Primer sequences are listed in Supplementary Figure S3B.

### RT-qPCR

K562 cells were maintained in RPMI supplemented with 10% FBS and 2 mM glutamine at 37°C in 5% CO_2_. Approximately 2.5 × 10^5^ cells were transfected with 1 µg of GFP, GFP-BCL11AXL or GFP-BCL11AXL (L319A/Y656A/W659A) using the Neon electroporation transfection system (Invitrogen) with the following parameters, 1350 v 10 ms, 4 pulses. At 48 h post transfection, Bgl3 and γ-globin gene transcripts were measured by RT-qPCR, which was carried out as described previously (33) using primers as described in Supplementary Figure S3C. GAPDH transcript levels were used as the reference gene, and assays were performed in triplicate.

## RESULTS

### F/YSXXLXXL/Y motifs mediate interaction of BCL11A with COUP-TFII LBD

**Figure 1.**
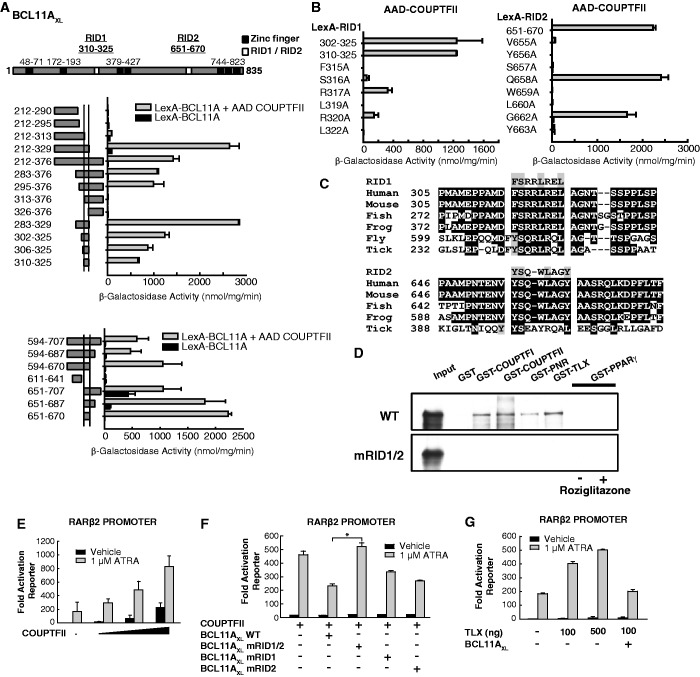
Novel signature motifs required for interaction of BCL11A-XL with COUP-TFII and related NRs. (**A**) Yeast two-hybrid interaction assays mapping the minimal sequences in BCL11A-XL that are required to bind the COUP-TFII LBD. LexA DBD-BCL11A fusion constructs were expressed in the L40 reporter strain either alone (black bars) or in conjunction with AAD-COUP-TFII LBD (grey bars). Reporter activity in cell-free extracts is measured as β-galactosidase activities (nmoles/min/mg protein). The data shown represent the mean of triplicate assays, and error bars represent the standard error of the mean. A schematic representation of BCL11A-XL protein is shown above the chart: black boxes represent Kruppel-like zinc fingers; white boxes represent the minimal COUP-TFII-interacting regions RID1and RID2. Amino acid numbers for the boundaries of RID, RID2 and zinc finger motifs are indicated. (**B**) Yeast two interactions of AAD-COUP-TFII LBD with RID1, RID2 and mutants thereof. (**C**) Alignment of BCL11A RID1 and RID2 sequences conserved in human, mouse, zebrafish, frog (*Xenopus laevis)*, fly (*Drosophila melanogaster)* and tick (*Ixodes scapularis*). (**D**) GST pulldown experiment showing interaction of *in vitro* translated ^35^S-labelled FLAG-BCL11A-XL wild type or RID1/RID2 double mutant (L319A/Y656A/W659A) with GST or GST-NR LBDs. For GST-PPARγ, binding was assessed in the presence of 1 µM of the agonist roziglitazone (+) or vehicle (−). (**E–G**) Reporter assays in transfected U2OS cells showing effect of BCL11A proteins on retinoic acid (ATRA)-induced activation of a RARβ2-luciferase. Reporter values were obtained after 24 hours exposure of transfected cells to 10^−6^ M ATRA (grey bars), or vehicle (black bars). Mean luciferase values from triplicate assays are shown, and error bars indicate the standard error of the mean.

BCL11A has been previously reported to contain two distinct regions (264–378 and 602–776) that can bind COUP-TFII proteins (7). To identify the interacting sequences more precisely, yeast two-hybrid (Y2H) mapping experiments were performed. Expression data for all two-hybrid constructs and confirmation of the lack of intrinsic reporter activation activities are provided in Supplementary Figures S1 and S2. Consistent with the previous report, the BCL11A sequences 212–376 and 594–707 were both capable of binding to the COUP-TFII LBD (144–414) independently of any added ligand (Figure 1A). Extensive deletion mapping precisely identified two short sequences (310–325 and 651–670; termed here RID1 and RID2) that were sufficient to bind the COUP-TFII LBD (Figure 1A).

To identify residues within RID1 and RID2 that are required for COUP-TFII LBD binding, alanine scan mutagenesis was performed. As show in Figure 1B, the substitutions F315A, S316A, L319A or L322A abrogated the binding of RID1 to COUP-TFII LBD. In contrast R317A and R320A retained substantial COUP-TFII binding, albeit that the reporter activity was 5-fold lower, indicating these residues are less critical for complex formation. Thus, the core sequence FSXXLXXL appears to be essential for binding of the RID1 sequence to COUP-TFII LBD. Similarly, V655A, Y656A, S657A, W659A, L660A and Y663A substitutions in RID2 prevented its interaction with COUP-TFII LBD whereas Q658A and G662A had no deleterious effect on binding in yeast two-hybrid assays (Figure 1B). This indicates that the sequence motif VYSXWLXXY is required for the interaction of RID2 with COUP-TFII. Both RID1 and RID2 are well conserved in BCL11A homologues across species (Figure 1C), and also within the closely related BCL11B gene (Figure 4A), and are predicted by the PSIPRED (http://bioinf.cs.ucl.ac.uk) algorithm to form amphipathic α-helices similar to the LXXLL (1) and CoRNR box motifs (3). Thus, we conclude that a core α-helical sequence motif fitting the consensus [F/Y]SXXLXX[L/Y] is important in mediating the interaction of BCL11A with the COUP-TFII nuclear receptor.

To confirm that RID1 and RID2 are both necessary and sufficient for the interaction of BCL11A with COUP-TFII, we introduced substitution mutations (L319A/Y656A/W659A) in full-length FLAG-BCL11A-XL that were predicted to prevent COUP-TFII binding based on the yeast two-hybrid data. We then tested the effect of these mutations on the interaction of *in vitro* translated, isotopically labelled full-length BCL11A-XL proteins with GST fusion proteins containing the LBDs of COUP-TFI/NR2F1 and COUP-TFII/NR2F2. We also included GST-LBD proteins consisting of the LBDs of TLX/NR2E1 and PNR/NR2E3, which are the close relatives of the NR2F subfamily, and PPARγ/NR1C3 whose sequence is more divergent. As shown in Figure 1D, interactions were detected between wild type BCL11A-XL and GST-COUP-TFI, GST-COUP-TFII, GST-TLX and GST-PNR proteins, suggesting BCL11A-XL binds directly to these LBDs. In contrast, the double mutant (mRID1/2) failed to bind to NR2E/F LBDs under similar conditions. No interaction of the wild-type BCL11A-XL was detected with controls i.e. GST alone, or GST-PPARγ LBD in the presence or absence of the agonist roziglitazone (Figure 1D). These results suggested that BCL11A-XL may undergo selective interactions with different members of the NR family, and that the RID1 and RID2 sequences are required for this function.

To assess the role of RID1 and RID2 sequences in promoting gene repression by COUP-TFII/BCL11A-XL complexes, reporter assays were performed using a RARβ2 promoter reporter. The RARβ2 gene proximal promoter contains binding sites for both RARs and COUP-TFs, which have been shown to cooperate to activate expression of this gene in response to ATRA treatment (34). We confirmed that COUP-TFII can associate with the RARβ2 proximal promoter sequences containing the COUP-TFII binding site in EMSA assays (Supplementary Figure S4A). Consistent with this, the RARβ2 reporter was induced by ATRA treatment due to the action of endogenous RARs, and was further activated by ectopic expression of COUP-TFII (Figure 1E). However, co-expression of COUP-TFII with wild-type BCL11A-XL protein repressed RARβ2 reporter activity. The repressive effect of BCL11A-XL on the RARβ2 reporter was inhibited by disruption of both RID1 and RID2 motifs, whereas only partial rescue was achieved by disruption of RID1 or RID2 alone (Figure 1F). Thus, physical interaction of BCL11A-XL with COUP-TFII via RID1 and RID2 motifs appears to be important for its corepressor function on the RARβ2 promoter. TLX has also been reported to activate the RARβ2 promoter in response to ATRA (35). Reporter assays confirmed that TLX-mediated activation of the RARβ2 reporter gene in response to ATRA is repressed by BCL11A-XL to a similar extent as that observed for COUP-TFII (Figure 1G). Thus, the ability of by NR2E/Fs to recruit BCL11A appears to be important for their transcription regulatory functions.

### RID1 and RID2 motifs are required for BCL11A-mediated repression of human γ-globin and Bgl3 genes

**Figure 2.**
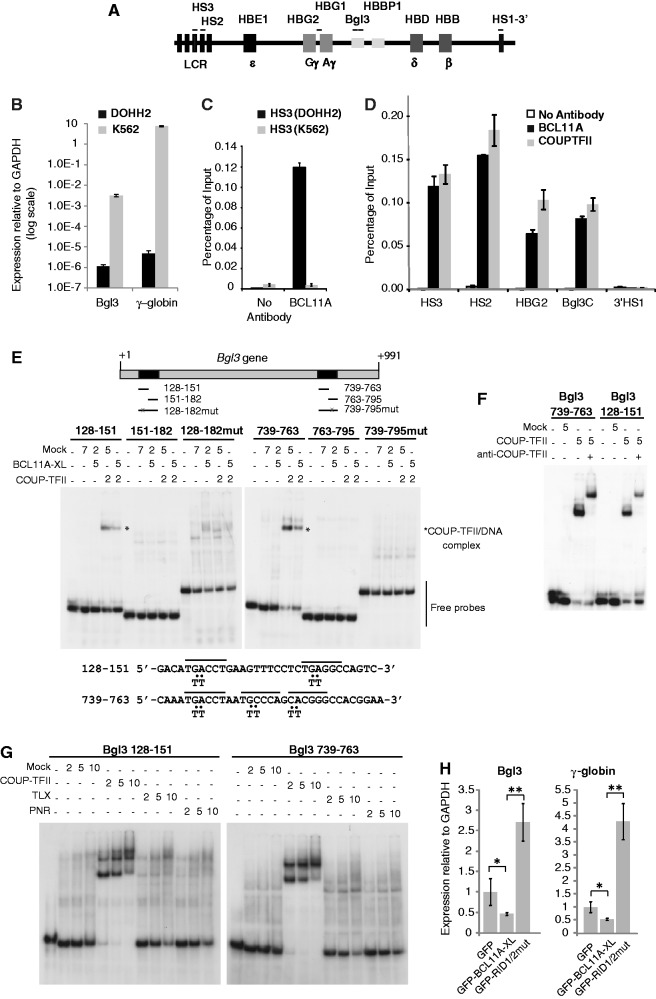
COUP-TFII binding sites in the Bgl3 locus, and requirement of RID1/RID2 for BCL11A-mediated repression of human γ-globin and Bgl3 genes. (**A**) Schematic representation of the human β-globin locus (not to scale) with boxes indicating the LCR and the embryonic (ε), fetal (Gγ and Aγ) and adult (δ and β) globin genes. The putative lncRNA Bgl3 and the pseudogene HBBP1 are also represented. Approximate regions amplified by PCR are indicated by lines over the boxes, including DNAse-I hypersensitive sites in the LCR (HS2 and HS3) and downstream region (HS1-3′). (**B**) Relative expression of Bgl3 and γ-globin transcripts (relative to GAPDH) in DOHH2 and K562 cells. The data are shown on a log scale as averages of triplicates and the error bars represent the standard deviations. (**C**) Chromatin IP qPCR revealing detection of BCL11A proteins at the HS3 site in the globin LCR in DOHH2 cells, but not K562 cells. Control (no antibody) is shown for comparison. The data shown are a representative experiment showing the average of triplicates, with error bars indicating standard deviations. (**D**) ChIP qPCR detection of BCL11A or COUP-TFII proteins at the LCR HS2 and HS3 sites, the γ-globin promoter, the Bgl3 sequence, and the HS1-3’ site. (**E**) EMSA indicating the presence of COUP-TFII binding sites within the human Bgl3 sequence. Double-stranded DNA probes (end labelled with γ^32^P) were incubated with cell-free extracts of HEK293 cells expressing recombinant Nuclear Receptor or BCL11A-XL proteins, or mock transfected as indicated (as described in Materials and Methods). The Bgl3 transcribed region is represented schematically, and the relative positions of the probes used in the EMSA experiments are indicated. Free probes and specific COUP-TFII/DNA complexes are indicated on the image. The sequences of the probes which showed specific complex formation with COUP-TFII are represented below the gel image. Putative HRE half sites are overlined and sequence changes made to disrupt these half sites in ‘mutated’ probes are indicated. (**F**) EMSA supershift experiment to validate of the identity of COUP-TFII containing complexes using a COUP-TFII specific antibody as described in Materials and Methods. (**G**) Comparison of the abilities of COUP-TFII, TLX or PNR proteins to bind the indicated Bgl3 probes. (**H**) RT-qPCR experiments indicating the effects of GFP, GFP-BCL11A and GFP-BCL11A(RID1/2)mut on endogenous γ-globin and Bgl3 expression (relative to GAPDH) in transfected K562 cells. The experiment was performed in triplicate with error bars indicating standard deviations, and *P* values indicated (*P < 0.05) (**P < 0.005).

To support the above conclusions, we assessed whether the RID1 and RID2 motifs are important for regulation of endogenous genes that are reported to be targets of COUP-TFII and BCL11A. Developmental regulation of the mammalian β-globin-like locus in erythroid cells is achieved as a consequence of functional interactions of BCL11A with transcription factors such as GATA factors, COUP-TFII, SOX6 and NF-Y (13,14,36,37). The switch from fetal to adult globin gene expression requires BCL11A, which acts to repress the fetal-specific γ-globin genes (Aγ and Gγ) (13,36,38–40). Similarly, COUP-TFII has been shown to repress γ-globin gene expression (37,41–43). BCL11A was also reported to repress the expression of the Bgl3 (AY034471) sequence, a series of non-coding transcripts located within the globin γδ-intergenic region (Figure 2A), which is co-regulated with the γ-globin genes (38). We therefore set out to find evidence for co-recruitment of COUPTF-II and BCL11A to regulatory sites in the globin locus, and to assess whether disruption of BCL11A/COUP-TFII complexes would impact on transcription of γ-globin and Bgl3.

To select a suitable cell line model, we assessed a number of leukaemia and lymphoma cell lines for expression of BCL11A-XL protein. Western blots indicated that BCL11A-XL is robustly expressed in the B-cell lymphoma DOHH2 cells and U937 myeloid leukaemia cells, whereas expression levels are very low in the erythroleukaemia cell line K562 and the promyelocytic leukaemia HL60 cell line (Supplementary Figure S5) (13,37,40). Consistent with current models that BCL11A functions as a repressor of fetal globin gene expression, very high levels of γ-globin and Bgl3 transcripts were detected in K562 cells using RT qPCR (Figure 2B). By contrast, γ-globin and Bgl3 transcripts were detected at extremely low levels (by several orders of magnitude) in the DOHH2 cell line (Figure 2B), which expresses relatively high levels of BCL11A-XL protein.

We next examined the association of BCL11A and COUPTFII proteins with key regulatory sites within the globin locus (depicted schematically in Figure 2A). Chromatin IP experiments confirmed that BCL11A protein was detected at DNAse hypersensitive site 3 (HS3) within the locus control region (LCR) in DOHH2 cells, but not in chromatin prepared from K562 cells (Figure 2C). Moreover, ChIP experiments using DOHH2-derived chromatin confirmed the presence of both BCL11A and COUP-TFII at the HS2 and HS3 sites of the LCR, the globin promoter region, the Bgl3 region, but not at the DNAse hypersensitive site 3′ to the locus (HS1-3′) (Figure 2D). In contrast, we did not detect BCL11A-XL at these sites in chromatin prepared from K562 cells, under similar experimental conditions (data not shown). These data are consistent with the potential presence of BCL11A/COUP-TFII complexes at key regulatory sites within the globin locus, and provide the first evidence that COUP-TFII may be involved in regulating the expression of the putative lncRNA Bgl3.

Analysis of the Bgl3 sequence for potential NR binding motifs highlighted two distinct regions containing consensus AGGTCA half sites, which were confirmed as COUP-TFII binding sites in EMSA assays (Figure 2E). Mutation of these half-sites disrupted the formation of COUP-TFII/DNA complexes (Figure 2E), while the presence of COUP-TFII in the complexes was confirmed by antibody supershifts (Figure 2F). These binding sites in Bgl3 appeared to be specific for COUP-TFII, as neither TLX/NR2E1 nor PNR/NR2E3 were capable of strong binding to these sequences in the EMSA assays (Figure 2G). In contrast to COUP-TFII, BCL11A did not produce strong shifts of EMSA probes, although at higher concentrations of BCL11A rather diffuse mobility shifts of Bgl3 probes were observed (Supplementary Figure S6A). Although the mobility of the specific COUP-TFII/DNA complexes was altered by BCL11A, suggesting potential interaction of COUP-TFII and BCL11A *in vitro*, we did not observe stable COUP-TFII/BCL11A/DNA complexes using this assay (Supplementary Figure S6A and B). Similarly weak binding of BCL11A proteins to the γ-globin promoter sequence in EMSA assays has been reported (37).Nonetheless, our results are consistent with reports that BCL11A can be recruited to the Bgl3 locus, and further suggest a role for COUP-TFII/BCL11A complexes in regulating Bgl3 transcription.

To assess the effect of RID1/2 mutations on the expression of Bgl3 and γ-globin expression, GFP-tagged versions of wild type BCL11A-XL or the RID1/2 mutant were ectopically expressed in K562 cells. The wild type GFP-BCL11A was able to repress expression of the endogenous Bgl3 and γ-globin genes (Figure 2H). In contrast, expression of the GFP-BCL11A-XL RID1/2 mutant in K562 cells resulted in a significantly enhanced expression of γ-globin over control (Figure 2H), suggesting a dominant negative effect such as sequestration of other co-repressors. Taken together our results indicate that repression of endogenous γ-globin and Bgl3 genes by BCL11A is dependent on the functionality of the RID1 and RID2 motifs, and thus provides strong evidence for a role for COUP-TFII in regulating expression of the Bgl3 locus.

### BCL11A RID1 and RID2 motifs are highly selective for the NR2E/F subfamily

**Figure 3.**
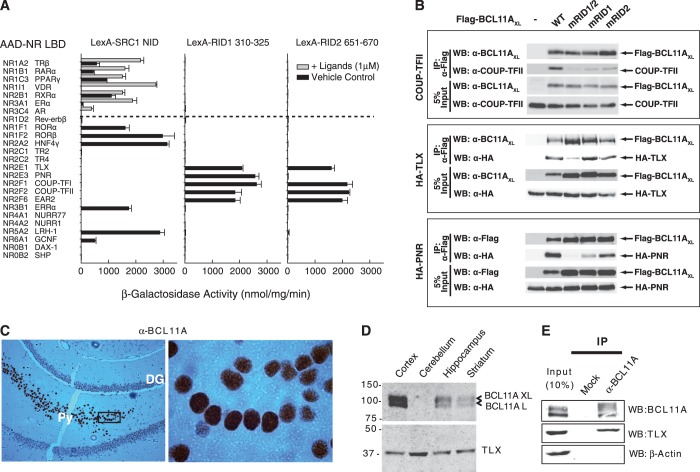
Selective nuclear receptor binding by BCL11A RID1 and RID2 motifs. (**A**) Yeast two-hybrid interactions of VP16 AAD-fused NR-LBDs with LexA DBD-fused SRC1 NID 489-827 (containing LXXLL motifs), or BCL11A RID1 and RID2 sequences as indicated. The results shown represent the mean reporter (β-galactosidase) activity ± standard error of triplicate experiments. Cognate ligands if known were added (grey columns), whereas vehicle only or no ligand is represented by black columns. (**B**) Co-immunoprecipitation of recombinant NR/BCL11A complexes. HEK293 cells co-transfected with COUP-TFII, HA-TLX or HA-PNR expression vectors in combination with FLAG-BCL11A-XL (WT), or the mutants mRID1/2 (L319A/Y656A/W659A), mRID1 (L319A) or mRID2 (Y656A/W659A). FLAG-tagged proteins were immunoprecipitated with anti-FLAG beads and associated proteins detected using the indicated antibodies. (**C**) Immunohistochemical staining of BCL11A proteins showing strong staining in the pyramidal cell layer (Py) extending from CA1 to the dentate gyrus (DG). Right panel shows magnification of the boxed region showing nuclear staining of BCL11A. (**D**) Immunodetection of TLX and BCL11A proteins in mouse brain tissue homogenates as indicated. (**E**) Co-immunoprecipitation of endogenous TLX and BCL11A proteins from adult mouse cerebral cortex tissue extract.

To determine whether the RID1 and RID2 sequences mediate binding to other NRs, we assessed interactions of LexA-RID1 and LexA-RID2 with a panel of 25 different VP16 AAD-NR LBD fusions in Y2H assays. The nuclear receptor interaction domain (NID) of the human SRC1 (489–827), containing three LXXLL motifs that mediate binding to liganded NR LBDs (1), was used for comparison. Cognate ligands (1 µM) were added to yeast transformants cultured overnight prior to preparation of cell-free extracts for reporter assays. As shown in Figure 3A, the SRC1 NID displayed strong ligand-stimulated interactions with NR1A2/TRβ, NR1B1/RARα, NR1C3/PPARγ, NR1I1/VDR, NR2B1/RXRα, NR3A1/ERα and NR3C4/AR, as we have previously reported (29,44). In addition, strong ligand-independent interactions were observed with the orphan receptors NR1F1/RORα, NR1F2/RORβ, NR2A2/HNF4γ, NR3B1/ERRα, NR5A2/LRH1 and NR6A1/GCNF (Figure 3A). Similar observations of SRC1 NID binding to these NR LBDs have been reported in the literature or protein interaction databases (e.g. BioGRID), with the exception of GCNF LBD, which, to our knowledge, was not previously reported to bind SRC1. We did not detect significant interactions of the SRC1 NID with NR0B1/DAX-1, NR0B2/SHP, NR1D1/Rev-erbβ, NR2C1/TR2, NR2C2/TR4, NR2E1/TLX, NR2E3/PNR, NR2F1/COUP-TFI, NR2F2/COUP-TFII, NR2F6/EAR2, NR4A1/Nurr77 or NR4A2/NURR1 under these conditions. In contrast, RID1 and RID2 sequences failed to bind any of the liganded receptor LBDs tested, or any orphan receptor LBD that showed an ability to interact with SRC1 (Figure 3A, middle and right panels). However, RID1 displayed strong binding to the LBDs of NR2E1/TLX, NR2E3/PNR, NR2F1/COUP-TFI, NR2F2/COUP-TFII and NR2F6/EAR2. Similarly, RID2 showed strong binding to NR2E1/TLX, NR2F1/COUP-TFI, NR2F2/COUP-TFII and NR2F6/EAR2, but failed to interact with the photonuclear-specific receptor NR2E3/PNR in these experiments. These results demonstrate a remarkable selectivity of BCL11A RID1 and RID2 motifs for the NR2E and NR2F subfamily of receptors.

To confirm that the observed interactions of RID1 and RID2 with NR2E and NR2F proteins in yeast reflect interactions of full-length proteins in mammalian cells, we performed co-immunoprecipitation experiments. HEK293 cells were transiently transfected with expression vectors for FLAG-BCL11A wild-type protein, RID1 mutant (L319A), RID2 mutant (Y656A/W659A) or a RID1/2 double mutant (L319A/Y656A/W659A) in combination with haemagglutinin (HA)-tagged TLX or PNR, or untagged COUP-TFII proteins. Co-immunoprecipitation experiments confirmed that wild-type BCL11A-XL was able to form complexes with COUP-TFII, TLX and PNR in HEK293 cells (Figure 3B), confirming the yeast two-hybrid results. Moreover, interactions with all three NRs were abrogated or reduced by the double RID1/RID2 mutation (Figure 3B), confirming the contribution of these motifs to establishing the observed interactions. The loss of either RID1 or RID2 reduced the ability of BCL11A-XL to bind COUP-TFII, suggesting both motifs may be required to form bivalent contacts with homodimer or heterodimer complexes of COUP-TFII. TLX on the other hand was more sensitive to mutations in RID2, as RID1 mutation (L319A) alone had little effect on its ability to form a complex with BCL11A-XL under these conditions. In contrast, mutation of RID2 had no deleterious effect on PNR binding, suggesting that only RID1 is required for interaction with this nuclear receptor, consistent with the yeast two-hybrid data (Figure 3A). Thus, these data confirm that NR2E/F subfamily members make selective contacts with BCL11A proteins through RID1/RID2 motifs, albeit with differing sensitivities to the ablation of either motif.

TLX is an important regulator of stem cell fate in forebrain and retina (45), and defects in its expression are associated with behavioural abnormalities, blindness and glioblastoma as reviewed in (46). In addition to its role in development, TLX expression has been detected in neural stem cells in adult brain within cells lining the subventricular zone, and the subgranular layer of the dentate gyrus, as well as in striatum and cortex (47). PNR expression in the adult is restricted to the photoreceptor cell layer of the retina (48). Previous studies have reported the detection of the BCL11A transcripts in both developing and adult murine brain (24). To examine BCL11A protein expression in brain, we performed immunohistochemical staining on mouse brain tissue sections using an antibody that detects both the L and XL isoforms. Strong expression of BCL11A was detected in cortical regions and also in pyramidal cell layers in the CA1 and CA3 regions of the hippocampus. BCL11A protein is localized to the nucleus in these cells, e.g. as shown for the pyramidal layer of the hippocampus (Figure 3C). Western blots on tissue homogenates confirmed the expression of BCL11A in mouse hippocampus and cortex, with lower levels detected in the striatum and very low levels in the cerebellum (Figure 3D). In contrast, TLX protein was detected in all four of these regions (Figure 3D). Importantly, immunoprecipitation of BCL11A proteins from cortex revealed co-precipitation of TLX but not the β-actin control (Figure 3E), confirming the existence of endogenous complexes of TLX and BCL11A *in vivo*. We also attempted to detect PNR/BCL11A complexes in whole eye homogenates from adult mice. However, although PNR was readily immunoprecipitated from these extracts, we were unable to detect expression of BCL11A in the eye (data not shown), suggesting that PNR/BCL11A interactions may occur only during embryo development.

### RID interaction requires the F221 residue in Helix 3 of the COUP-TFII LBD

**Figure 4.**
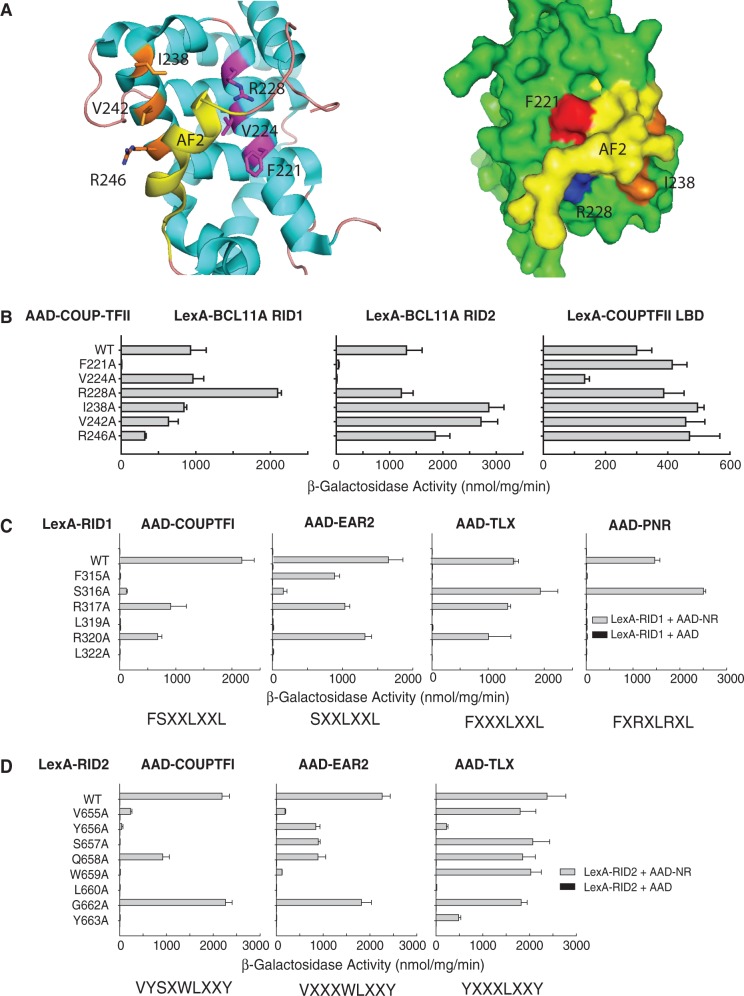
Probing the binding interface between NR2E/Fs and BCL11A. COUP-TFII LBD Helix 3 residues facilitate docking of RID1 and RID2: (**A**) Cartoon representation (left panel) of the COUP-TFII LBD (PDB: 3CJW) (49) generated using PyMol open source molecular visualization tool. The AF2 helix is shown in yellow. Key residues in Helix 3 (magenta) and Helix 5 (orange) are indicated. Right panel shows a surface representation of the COUP-TFII LBD complex highlighting the exposed phenylalanine F221 (red) and R228 (blue) which hydrogen bonds to the AF2 helix. In this view, V224 is buried beneath the AF2 helix. (**B**) Yeast two-hybrid interactions of LexA-BCL11A RID1 and LexA-BCL11A RID2 with AAD-COUP-TFII LBD wild type or mutant proteins as indicated. The right panel shows the effect of these mutations on heterodimer formation with LexA COUP-TFII LBD. The results shown represent the mean reporter (β-galactosidase) activity ± standard error of triplicates. Differential sequence requirements for binding of RID1 and RID2 to NR2Es and NR2Fs: Yeast two-hybrid interactions of (**C**) LexA-BCL11A RID1 and (**D**) LexA-BCL11A RID2 alanine scan mutant series in combination with AAD-NR LBD constructs as indicated. The results shown represent the mean reporter (β-galactosidase) activity ± standard error of triplicates. The sequence motif required for the binding of each NR LBD is indicated below the graph.

We noted from the structure of the COUP-TFII LBD (PDB:3CJW) that residues in Helix 3 (F221, V224, R228) and Helix 5 (I238, V242, R246) are likely to be positioned to come in contact with α-helices in docking cofactors (Figure 4A). We therefore generated AAD-COUP-TFII LBD substitution mutants for Y2H assays. As shown in Figure 4B, substitution of F221 with alanine completely abrogated the binding of both RID1 and RID2, indicating that this residue is essential for binding to the F/YSXXLXXL/Y helices. The ability of the F221A mutant to form homodimers with wild-type COUP-TFII LBD was not affected, showing that this mutation is unlikely to perturb the overall structure of the domain (Figure 4B). While the V224A mutant appeared unable to bind RID2, RID1 binding appeared unaffected (Figure 4B). However, western blots indicated that this mutant was expressed at a 2-fold lower level than other constructs in this series (Supplementary Figure S2C). Consistent with this, the V224A also showed reduced reporter activation due to homodimer formation (by about 2.5-fold), which we attribute to its slightly reduced expression level. Mutated LBDs with replacements of I238, V242 or R246 in Helix 5 (or K362 in Helix 10, data not shown) retained substantial ability to bind RID1 or RID2, albeit that binding of RID1 by the R246A mutant was reduced. This suggests that these residues are not essential for binding of COUP-TFII LBD to BCL11A. Substitution of R228, which is the equivalent of the conserved lysine in the charge clamp contacting LXXLL motifs in liganded NRs, did not disrupt the interaction of the COUP-TFII LBD with RID1 or RID2. Indeed, reporter activation due to interaction of the R228A mutant with RID1 was enhanced in comparison with the wild-type LBD (Figure 4B). As R228 is predicted to stabilize the orientation of H12 within the cofactor docking site (49), it is possible that disruption of this salt bridge might facilitate H12 displacement by cofactors. We conclude from these experiments that Helix 3 is likely to form part of the RID1 and RID2 docking site on the COUP-TFII LBD surface. While F221 appears critical for binding RID1 and RID2, V224 may be essential for RID2 binding only. This is suggestive of subtle differences in how the two motifs contact COUP-TFII.

In the ‘auto-inhibited’ structure of the COUP-TFII LBD, the activation function-2 (AF2) helix occupies the cofactor binding groove on the LBD surface (Figure 4A). It has been postulated that binding of COUP-TFII to retinoic acid (ATRA) ligand would alter the LBD structure (49) thus facilitating its interaction with cofactors. We did not observe any effect of ATRA on the binding of coactivator or corepressor peptides in two-hybrid assays, or on interactions of full-length proteins *in vitro* (data not shown), although we cannot rule out the presence of low levels of endogenous ATRA in these assays. However, deletion of the COUP-TFII AF2 helix (Δ393–414) resulted in a complete loss of interaction with RID1 and RID2, and a failure to form heterodimers with full-length LBD (Supplementary Figure S2D), suggesting that the AF2 helix is required to stabilize the COUP-TFII LBD structure.

### Differential requirements of COUP-TFs, TLX and PNR for RID1 and RID2 sequences

**Figure 5.**
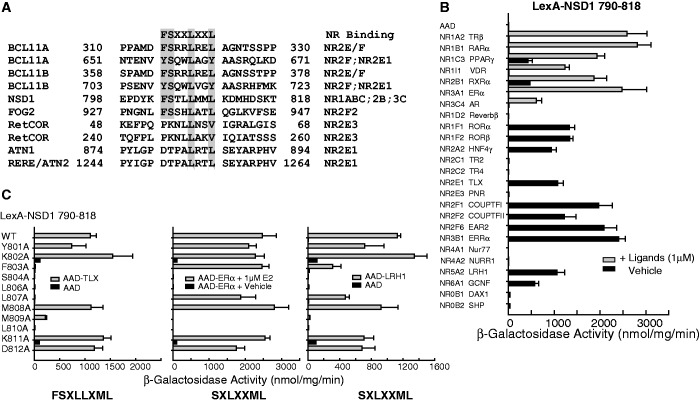
A functional FSXXLXXL motif in NSD1. (**A**) Sequence alignment showing the presence RID1/RID2 and ATRO box sequences in NR2-binding cofactors. The sequences are based on human Refseq entries in NCBI Protein or Gene databases. Conserved FS and L residues are shaded. Predicted alpha-helicity of the BCL11A RID1 sequence is shown above the alignment. (**B**) Yeast two-hybrid interactions of LexA DBD-fused NSD1 790–818 with AAD-NR-LBD constructs as indicated. Cognate ligands if known were added (grey columns), whereas vehicle only or no ligand is represented by black columns. The results shown represent the mean reporter (β-galactosidase) activity ± standard error of triplicate experiments. (**C**) Binding of LexA NSD1 790–818 wild type or mutants to AAD-TLX, AAD-ERα, AAD-LRH1 LBDs. The sequence of the NSD1 NID+L region (790–818) is indicated, and the sequence motif required for binding to different NR LBDs is indicated above the bar charts.

To determine whether members of the NR2E/F subfamily make similar contacts with the RID1 and RID2 motifs, yeast two-hybrid assays were performed to assess the interactions of NR2E/F receptors with the mutant RID1 and RID2 constructs described in Figure 1B. As shown in Figure 4C and D, COUP-TFI showed a similar pattern of interactions with RID1 and RID2 mutants as found for COUP-TFII (Figure 1B), showing a requirement for FSXXLXXL in RID1 (Figure 4C), and VYSXWLXXY in RID2 (Figure 4D). The EAR2/NR2F6 LBD showed only 2-fold lower reporter activation due to interactions with F315A, suggesting this residue is less critical for its recruitment of RID1 (Figure 4C). Similarly, EAR2 engagement of RID2 was somewhat different to COUP-TFI/II, as alanine substitution of Y656 or S657 had only a minor effect on reporter activation. This suggests a requirement for VXXXWLXXY in RID2 to bind EAR2. TLX binding to RID1 did not require S316A (Figure 4C) and similarly S657 in RID2 was dispensible for TLX recruitment (Figure 4D). Combined with other interaction data, this suggests that TLX requires FXXXLXXL and YXXXLXXY in RID1 and RID2, respectively. Interestingly, the SANT domain repressor proteins Atrophin-1 (ATN1) and RERE/ATN2, which can bind TLX (50) and PNR (51), contain a motif termed the ATRO box motif that is required for interaction with TLX (50), This sequence (LXXL) shows partial overlap with the C-terminal portions of RID1 and RID2 (Figure 5A).

The interaction of PNR with BCL11A was somewhat distinct from other members of the NR2E/F subfamily, in that it was found to be dependent on RID1, but much less so on the integrity of RID2. Similar to COUP-TFI/II and TLX, binding of PNR to RID1 required F315, L319 and L322. However, unlike other NRs, PNR was also sensitive to mutation of R317 and R320 (Figure 4C). This result indicates a requirement for the sequence FXRXLRXL to promote stable interaction of BCL11A with PNR. As the RID2 motif lacks equivalent arginine residues at these positions, this might account for the apparent preference of PNR for RID1.

### Conservation of F/YSXXLXXL/Y motifs in NSD1 and other proteins

We have shown here that BCL11A RID1 and RID2 sequences mediate selective interactions with the NR2E/F subfamily, and that F/YSXXLXXL/Y motifs are conserved in vertebrate, insect and arthropod orthologs of BCL11A/B proteins (Figure 1C). Sequence similarity searches revealed the existence of similar motifs in other transcriptional regulators including the NR cofactor SOTOS overgrowth syndrome gene product NSD1 (Figure 5A). NSD1 is a SET domain lysine methyltransferase protein containing two regions that exhibit differential interactions with NRs (52). The sequence 738–788 was reported to bind to the LBD of RARα in the absence of ligand, whereas addition of ATRA disrupted this interaction (52), similar to the binding behaviour of corepressors such as NCOR and SMRT with RARs and other liganded NRs (3–5). In contrast, an adjacent region of the NSD1 sequence (termed NID+L) displayed binding properties typical of a coactivator in that it bound to the LBDs of RARα, RXRα, ERα and TRα, in a ligand-dependent manner. This function was mapped to a LXXLL-type sequence defined as the sequence NSD1 803-FXXLL-807 (52), which is coincident with the RID1-like sequence 803-FSXXLXXL-810 (Figure 5A). To test whether this sequence would facilitate interactions with NR2E/F proteins, as predicted by our findings, we performed two-hybrid assays. As shown in Figure 5B, the construct LexA-NSD1 790-818, which contains an RID1-like motif, showed strong ligand-dependent interactions with NR1B1/RARα, NR2B1/RXRα and NR3A1/ERα as reported previously, but also with NR1C3/PPARγ, NR1I1/VDR, NR1A2/TRβ and NR3C4/AR. Substantial constitutive interaction with RXRα and PPARγ occurred in the absence of exogenous ligand for the reasons previously outlined, consistent with similar results for SRC1 and MED1 NIDs (1,29,44). In addition, as observed for SRC1 NID, strong constitutive interactions were observed with the orphan receptors NR1F1/RORα, NR1F2/RORβ, NR2A2/HNF4γ, NR3B1/ERRα, NR5A2/LRH1 and NR6A1/GCNF, but not with NR0B1/Dax-1, NR0B2/SHP, NR1D2/Reverbβ, NR2C1/TR2, NR2C2/TR4, NR4A1/Nurr77, NR4A2/NURR1 or NR2E3/PNR (Figure 5B). However, unlike the SRC1 NID (Figure 3A), the NSD1 NID+L containing construct also showed strong interactions with NR2F1/COUP-TFI, NR2F2/COUP-TFII, NR2F6/EAR2 and NR2E1/TLX (Figure 5B). This result is consistent with the hypothesis that a FSXXLXXL sequence within NSD1 can mediate interactions with the NR2E/F subfamily. The failure of the NSD1 motif to interact with PNR LBD in these assays is also consistent with the observed differential sequence requirement of PNR e.g. for a FXRXLRXL sequence, as present in BCL11A proteins.

Our data indicate that short alpha helical motifs present in cofactors determine differential NR-binding selectivity. Although BCL11A and NSD1 both have FSXXLXXL sequences, BCL11A is highly selective for NR2E/F subfamily, whereas NSD1 shows a much broader range of interactions. We hypothesized that this differential selectivity may be due to the sequence context, which we have previously shown to be important in determination of NR selectivity (1,29,44). In contrast to the BCL11A RID1, the NSD1 NR-binding sequence is coincident with FXXLL and LXXML motifs, both of which are LXXLL variants that can potentially dock with ligand-dependent NRs. Thus, we reasoned that the NSD1 motif may contain structural features that are recognized by different NR subgroups. To test this, we undertook substitution mutagenesis of the NSD1 motifs to determine the effects on binding of NRs from different binding classes. As shown in Figure 5C, interaction of the NSD1 motif with the LBD of TLX binding required a FSXLLXML sequence, whereas binding to ERα or LRH1 LBDs required the sequence SXLXXML, which more resembles the LXXLL signature motif. These differential requirements for docking with different NR LBDs suggest that cofactor motifs have co-evolved with NR LBD sequences to achieve selective interactions. Thus, conserved alpha helical motifs present in NR cofactors have evolved features that accommodate their selective binding to different subsets of NRs within the superfamily.

## DISCUSSION

We have identified novel sequence motifs in the developmental co-repressor BCL11A that facilitate its selective interactions with members of the NR2E/F subfamily. These motifs, RID1 and RID2, bear resemblance to the LXXLL and LXXXLXXXI/L canonical motifs found in NR coactivators and corepressors, respectively. The BCL11A motifs are highly selective for the NR2E/F subfamily, in contrast with LXXLL motifs found in coactivators such as SRC1 (Figure 3A) and MED1 (data not shown), which do not show strong binding to these NRs. Our study also reports the interaction of TLX/NR2E1 and PNR/NR2E3 with the developmental co-repressor BCL11A.

**Figure 6.**
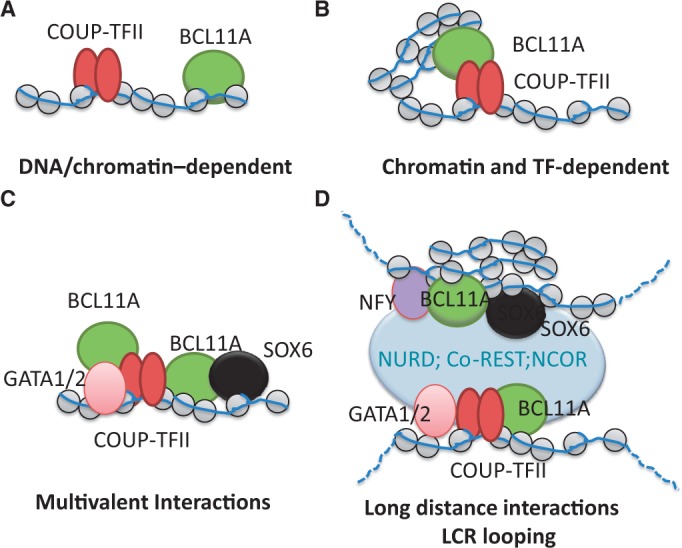
Schematic model showing possible modes of recruitment of BCL11A to regulatory sites in the globin locus, and the formation of functional complexes with COUP-TFII; (**A**) Direct, independent recruitment of both factors to DNA sequences or chromatin; (**B**) co-dependent recruitment through protein–protein interactions mediated by F/VSXXLXXL/Y motifs; (**C**) Multivalent interactions between different factors assembled *in situ*; (**D**) Long distance interactions involving transcription factors and chromatin regulator complexes e.g due to chromatin looping.

Interactions of BCL11A with COUP-TFII were known from previous studies, and there is evidence that these proteins cooperate to regulate expression of genes in the globin locus and are thus important in fetal to adult haemoglobin switching. Early studies described mutations within the human γ-globin promoter that are associated with the persistent expression of the γ-globin genes in adults, which were later shown to function as composite COUP-TFII/GATA binding sites (53). More recently, it has been reported that a non-coding sequence in the globin locus termed Bgl3 shows co-expression with γ-globins (40). Moreover, both of these transcripts have been shown to be regulated by BCL11A (13,40), although the function of the Bgl3 lncRNA remains to be determined. We have shown here that similar to COUP-TFII-dependent regulation of the γ-globin genes (37,42), the Bgl3 locus contains binding sites for COUP-TFII, confirmed in EMSA and ChIP qPCR assays, suggesting Bgl3 transcription is regulated by this factor. Indeed, using the K562 erythroleukemia cell line which contains only very low levels of BCL11A (Supplementary Figure S5), we showed that exogenous BCL11A-XL can repress expression of the Bgl3 and γ-globin genes, and that this is dependent on the functionality of the RID1 and RID2 motifs. These results indicate that interaction of BCL11A and COUP-TFII proteins is important for regulation of transcription within the globin locus. However, understanding the precise nature of these functional interactions is challenging, given that both factors may bind DNA/chromatin independently, co-dependently or via associations with a complex network of other transcription factors (GATA1/2, SOX6, NF-Y) and co-repressors (NuRD, HDACs, SIRT1, BCL6, Co-REST, NCoR) at globin gene regulatory sites, as shown schematically in Figure 6. Further studies will be required to determine the precise mechanisms involved.

TLX is a key stem cell fate regulator in the developing forebrain and eye (45,54). In adult mice, TLX is expressed in the subventricular zone of the lateral ventricle and subgranular layer of the dentate gyrus, both of which are regions associated with neurogenesis (45,55). Consistent with *in situ* hybridization studies on BCL11A transcripts (24), we detected substantial expression of BCL11A-XL and L isoforms in the hippocampus and cortex, and to a lesser extent in striatum (Figure 3C and D). Moreover, we were able to demonstrate that TLX and BCL11A proteins can be co-immunoprecipitated from extracts of adult mouse cerebral cortex (Figure 3E). This suggests that BCL11A may function as a corepressor in TLX-mediated gene regulation in adult neuronal tissues. While genes that are regulated by TLX/BCL11A remain to be identified, a previous study has shown that TLX can activate the RARβ2 promoter in response to ATRA (35). Reporter assays confirmed that TLX-mediated activation of the RARβ2 reporter gene in response to ATRA is repressed by BCL11A to a similar extent as observed for COUP-TFII (Figure 1F and G).

PNR expression in adult mammalian tissues is confined to photoreceptors in the retinal epithelial layer. Knockout studies have shown that PNR suppresses cone gene expression in photoreceptors, thus committing precursor cells to the rod lineage. PNR acts in conjunction with cone rod homeobox (CRX) and neural retina leucine zipper (NRL) to regulate rod and cone gene expression in a cell-specific manner, and appears to direct both positive and negative regulation (56). The mechanism by which this is achieved, and the cofactors that are involved remain unclear, although it has been reported that PNR can recruit Atrophins (51) and the Ret-CoR corepressor complex (57). Our findings indicate that PNR can also interact directly with BCL11A-XL, as demonstrated by yeast two-hybrid, *in vitro* studies and in transfected cells (Figures 1 and 3). However, as we did not detect expression of BCL11A -L or XL isoforms in the eye (data not shown), it remains to be established whether coexpression of BCL11A and PNR occurs in other tissues, such as in the developing embryo. Alternatively, it is possible that PNR interacts with BCL11B or other proteins containing RID1-related motifs.

Both TLX and PNR have been reported to bind the Atrophin/RERE proteins that function as transcription corepressors (50,51). Sequence alignments revealed that the F/YSXXLXXL/Y motifs identified in BCL11A show partial similarity to a motif defined in Atrophin/RERE proteins that mediates binding to TLX (Figure 5A), termed the ATRO box (50). Although saturation mutagenesis to define the ATRO box remains to be determined, it has been shown that the sequence LXXL is required for association of atrophins with TLX (50). It remains to be established whether atrophins are selective for PNR and TLX, or whether atrophins can form complexes with COUP-TFs or other NRs. The RNA helicase Ret-CoR/DHX30 was isolated in a yeast two-hybrid screen of human brain cDNAs for PNR-binding proteins (57). Like atrophins, Ret-CoR is a component of a HDAC complex, which was shown to act as a corepressor for GAL4-PNR-mediated transcription. The interaction of Ret-CoR with PNR was reported to be dependent on two copies of the sequence (LXXVI) (57), which has similarity to CoRNR box and ATRO box motifs. However, it is not been established if this protein can bind other NRs. Interestingly, while Ret-CoR/PNR complexes were shown to occur in the developing retina, expression of Ret-CoR in adult retinal tissues was very low (57). Thus interactions of RetCoR and BCL11A/B with PNR may be restricted to embryonic development.

Biochemical and structural studies have revealed that NR LBDs undergo conformational changes in response to agonist/antagonist binding, and that these changes regulate interactions with coactivators and corepressors. Crystal structures of apo- and holo-receptor LBDs have demonstrated the conformational flexibility of the AF2 helix in ‘activating’ or ‘repressive’ conformations of the LBD, and this positioning is thought to facilitate the docking of coactivators and corepressors [reviewed in (53,58)]. Both coactivator (LXXLL) peptides and repressor peptides occupy the same docking site, suggesting a mutually exclusive mechanism of interaction. Among the NR2E/F subfamily, only the crystal structure of the unliganded COUP-TFII monomer has been determined, and this shows the AF2 helix occupying the cofactor binding pocket on the LBD surface (49). Based on other LBD structures, this autoinhibitory conformation is likely to be refractory to cofactor binding. Thus, the question remains as to how corepressors and coactivators dock with COUP-TFII LBD. Our mutagenesis assays indicate that residues in Helix 3 that help to accommodate the AF2 helix in the ‘auto-inhibited’ LBD are involved in the binding of BCL11A while F221 is required to facilitate both RID1 and RID2, V224 appears to be essential for RID2 only. These results are suggestive of subtle differences in how RID1 and RID2 motifs dock with the COUP-TFII LBD, possibly involving different contacts with cofactor binding groove to displace the AF2 helix. This is reminiscent of the different binding mechanisms observed for the interaction of the ID1/CoRNR1and ID2/CoRNR2 motifs with the RAR LBD (59). Structural studies will be required to reveal how RID1 and RID2 sequences dock with NR2E/F LBDs.

Although COUP-TFs can synergize with coactivators to activate transcription in reporter assays (49) evidence for direct interactions of the COUP-TFs with the coactivators is lacking. In our hands, COUP-TFII LBD binding to full length proteins or LXXLL peptides derived from SRC1 or MED1/TRAP220 is not robust enough to be detected in Y2H or GST pulldown assays (Figure 3A and data not shown). Thus, the mechanism by which COUP-TFII activates transcription remains to be determined. It is possible that COUP-TFs may recruit coactivators indirectly via heterodimer partners, or that the observed positive effects of COUP-TFII on transcription are due to derepression mechanisms, such as sequestering corepressors away from target genes. Although the NR-binding LXXLL motifs in SRC1 (Figure 3) and MED1/TRAP220 (data not shown) appear to interact exclusively with ‘activating’ NRs, we have shown here that the NR binding motif of NSD1 has features that can promote its binding to both ‘activating’ and ‘repressive’ NRs. Interestingly, the failure of PNR to bind NSD1 FSXXLXXL motif indicates a more specific sequence requirement consistent with the mutagenesis data in Figure 4C. It remains to be investigated whether NSD1 forms complexes with NR2E/Fs *in vivo*, in particular in brain where both proteins are highly expressed.

In addition to BCL11A/B and NSD1, F/YSXXLXXL/Y motifs are found in other proteins including NRs, NR cofactors and other gene regulatory proteins (see Supplementary Figure S7). It remains to be established whether these motifs can promote direct interactions with NR2E/F subfamily members, although for some there is previous evidence that such complexes exist. For example, the zinc finger repressor Friend of GATA (FOG2) is known to interact with both GATA and COUP-TFII proteins to regulate globin gene expression in haematopoietic tissues (60,61). Intriguingly, the C-terminal sequence 848–1152, which mediates interaction with COUP-TFII, comprises a FSXXLXXL motif (Figure 5A). Further studies will be required to determine whether RID1/RID2 motifs are unique to the BCL11A/B and NSD1 proteins or are more widespread amongst cofactors for COUP-TFs, TLX and PNR.

In summary, we have defined a new signature motif selective for the NR2E/F subfamily and highlighted previously unknown roles for BCL11A/B and NSD1 proteins in the gene regulation by COUP-TFs, TLX and PNR. Moreover, our study provides new insights into how subtle variations in short sequences at the interface of NR/cofactor complexes determine selectivity.

## SUPPLEMENTARY DATA

Supplementary Data are available at NAR Online, including [62–67].

## FUNDING

Gordon Pillar Studentship from Leukaemia Lymphoma Research [06090 to C.M.C.]; studentship from the General Pharmaceutical Council (RPSGB) (to J.F.) via an Academic Excellence Award (to D.M.H.); Clinical Research Fellowship from Leukaemia Lymphoma Research [07064 to F.W.]; project grant from the Wellcome Trust [WT0845921Z to P.M.M.]; project grants from Leukaemia Lymphoma Research [0263], the Association of International Cancer Research [03-0214 to D.M.H.]; and Cancer Research UK [C1506/A11643] (in part). Funding for open access charge: University of Nottingham.

*Conflict of interest statement*. None declared.

## Supplementary Material

Supplementary Data
