# Supplementary material for: A signature motif mediating selective interactions of BCL11A with the NR2E/F subfamily of orphan nuclear receptors
Source: Nucleic Acids Res. 2013 Aug 23;41(21):9663–79. doi: 10.1093/nar/gkt761 (PMC3834829; doi:10.1093/nar/gkt761)
Supplement: Supplementary Data [file supp_41_21_9663__index.html]

A signature motif mediating selective interactions of BCL11A with the NR2E/F subfamily of orphan nuclear receptors — Supplementary Data 

# A signature motif mediating selective interactions of BCL11A with the NR2E/F subfamily of orphan nuclear receptors

## Supplementary Data

files

**Files in this Data Supplement:**

- Supplementary Data - pdf file
