# Supplementary material for: A signature motif mediating selective interactions of BCL11A with the NR2E/F subfamily of orphan nuclear receptors
Source: Nucleic Acids Res. 2013 Aug 23;41(21):9663–79. doi: 10.1093/nar/gkt761 (PMC3834829; doi:10.1093/nar/gkt761)
Supplement: Supplementary Data [file supp_gkt761_nar-01759-x-2012-File008.pdf]

SUPPLEMENTAL DATA

FIGURE S1

Expression of LexA-BCL11A fusion constructs in yeast

Western blots were used to confirm the expression of the LexA-BCL11A fusion proteins used in this study, using anti-LexA antibody as described in the Materials & Methods. In all cases we were satisified that the estimated molecular weight of the detected constructs was as expected, and that any differences in expression levels were not sufficient to account for changes in reporter activities due to Y2H interaction. If possible, related constructs were run on the same blot for comparison. However, if composite figures are shown, this is emphasised by spacing apart of images.

Fig S1A: RID1 deletion mapping constructs

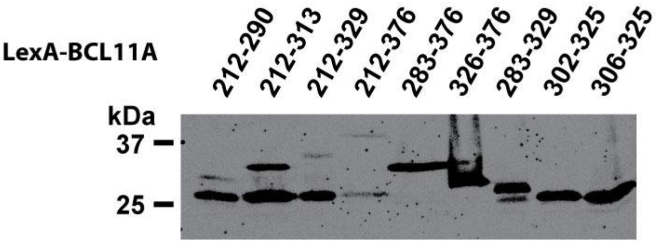

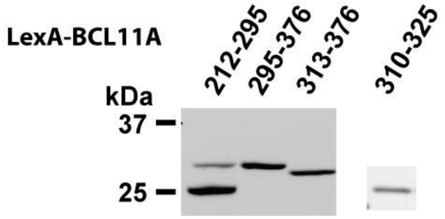

Fig. S1B RID2 deletion mapping constructs

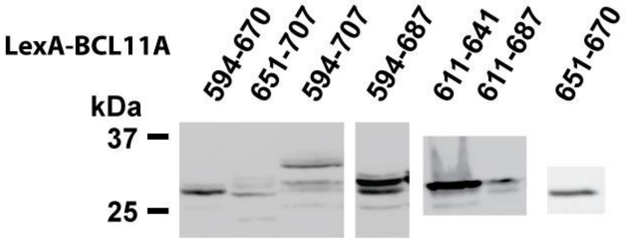

Fig. S1C RID1 Substitution Mutations

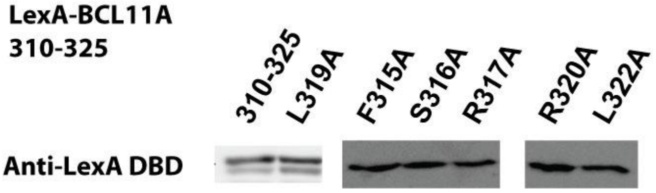

Fig. S1D RID2 Substitution Mutations

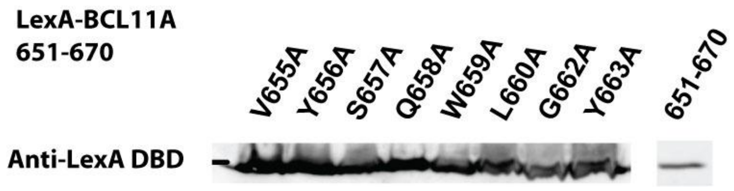
LexA-BCL11A
651-670
V655A
Y656A
S657A
Q658A
W659A
L660A
G662A
Y663A
651-670
Anti-LexA DBD

FIGURE S2
A&B. Expression and Activity of AAD-NR LBD fusion constructs in yeast

(A) Western blots were used to confirm the expression of the AAD (VP16 411-490)-NR LBD fusion proteins in *S.cerevisiae* L40 cell-free extracts, using anti-VP16 antibody as described in the Materials & Methods. In all cases we specifically detected recombinant proteins of the correct estimated molecular weight. To assess whether these recombinant proteins show any inherent ability to activate the lacZ reporter gene, AAD-NR LBD constructs were cotransformed with LexA DBD alone. As shown in (B), activation of the reporter due to to LexA alone in combination with AAD or any of the 25 AAD-NR constructs was negligible. Reporter activation due to interaction of AAD-NR LBDs with LexA-SRC1 NID is shown for comparison (SRC1 data already shown in Fig. 2A)

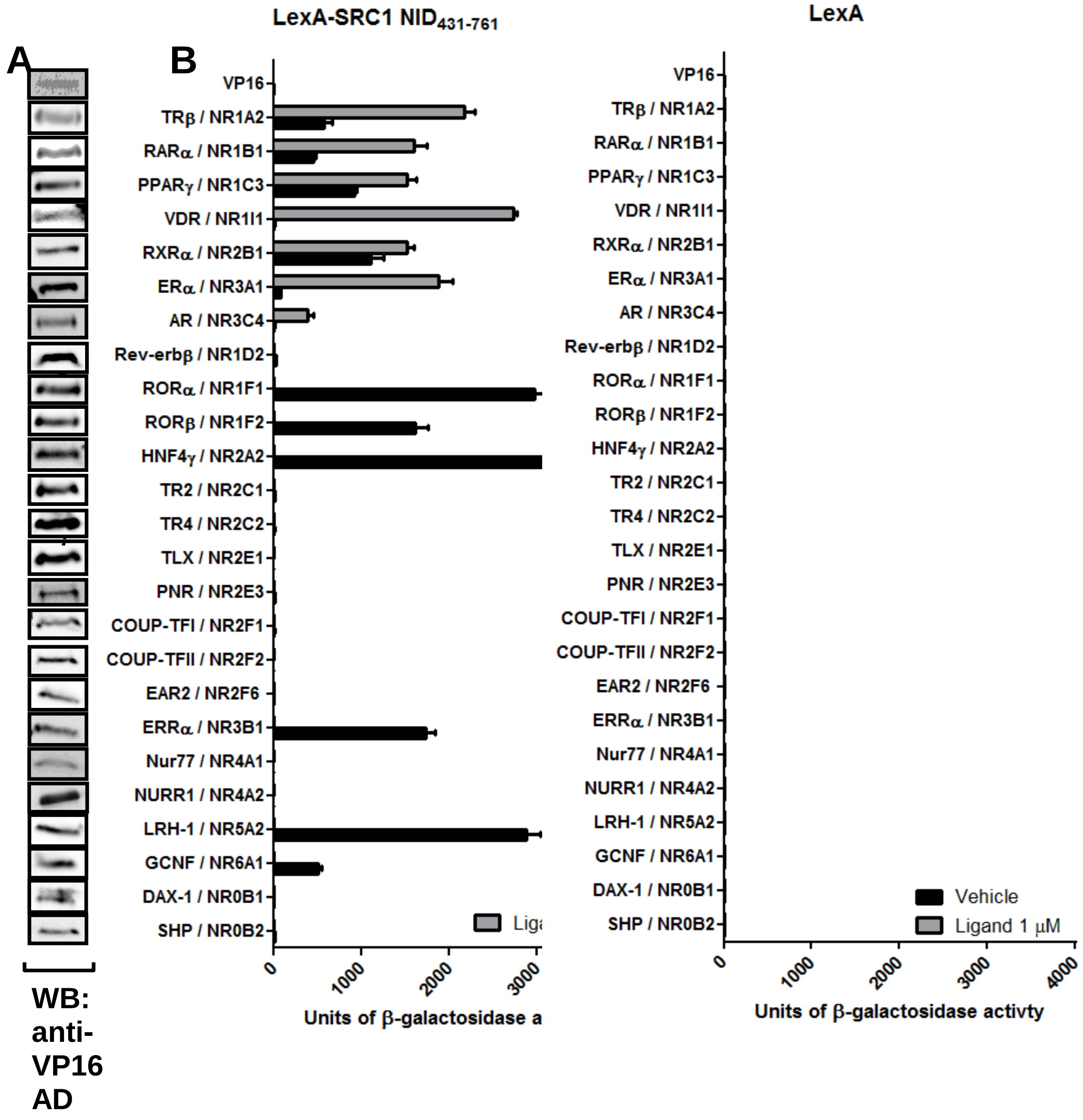

C. Western blot detection of AAD-COUP-TFII LBD mutants

Western blots detecting AAD-COUP-TFII LBD substitution mutants in *S.cerevisiae* L40 cell-free extracts, using anti-VP16 antibody as described in the Materials & Methods.

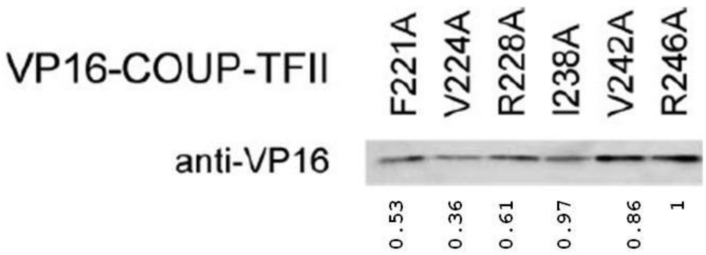

Quantitation relative to the highest expression level (set to 1) was performed using the Image j analysis tool.

D. Interaction of COUP-TFII with RID1 and RID2 is dependent on the AF2 helix

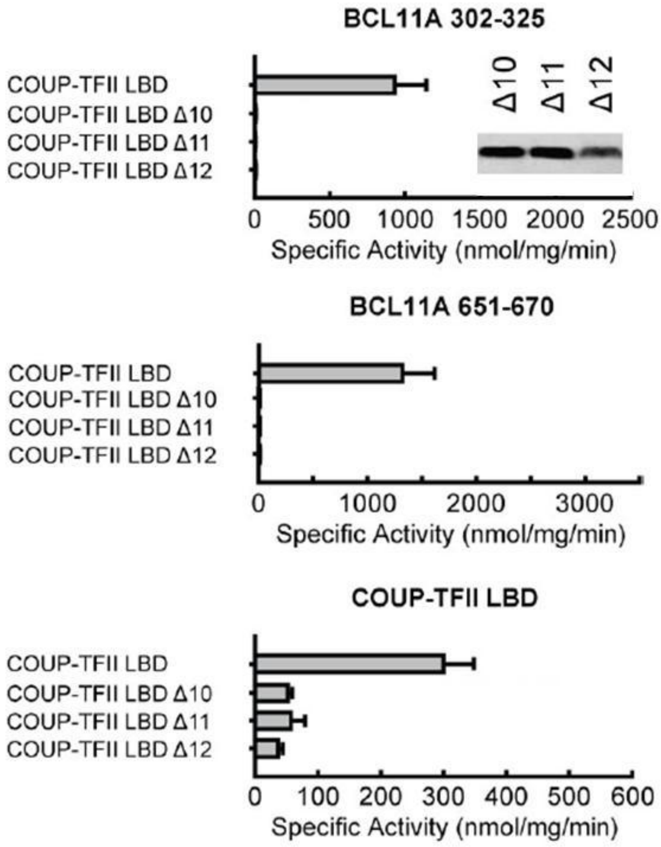

Yeast two-hybrid assays showing the effect of C-terminal deletions on the interaction of COUP-TFII LBD with the BCL11A RID1 and RID2 sequences. The Δ12 deletion refers to the AF2 helix Δ12 whereas Δ11 truncates helices 11 and 12, and Δ10 deletes helices 10,11,12 as based on the reported structure (ref. 49). Western blot detection of the mutants is shown in the inset.

FIGURE S3

A. Oligonucleotide Probes used for EMSA experiments

Sequences of the sense and antisense strand primers used in this study are listed below. Potential HRE half sites are underlined. Bases that have been mutated to disrupt HREs are highlighted in bold.

Bgl3 Sequences

+198/+231
5'-TTCCGTGGCCCGTGCTGGGCATTAGGTCATTT-3'
3'-AAATGACCTAATGCCCAGCACGGGCCACGGAA-5'

+231/+255
5'-TGTGTCTGGCACAGGCTTGATCTATA-3'
3'-TATAGATCAAGCCTGTGCCAGACACA-5'

+198/+255
5'-TTCCGTGGCCCGTGCTGGGCATTAGGTCATTTGTGTCTGGCACAGGCTTGATCTATA-3'
3'-TATAGATCAAGCCTGTGCCAGACACAAATGACCTAATGCCCAGCACGGGCCACGGAA -5'

+198/+255 mutant
5'-TTCCGTGGCCCG**AA**CTGG**AA**ATTAGG**AA**ATTTGTGTCTG**AA**ACAGGCTTGATCTATA-3'
3'- TATAGATCAAGCCTGT**TT**CAGACACAAAT**TT**CCTAAT**TT**CCAG**TT**CGGGCCACGGAA -5'

+812/+843
5'-GACTGGCCTCAGAGGAAACTTCAGGTCATGTC-3'
3'-GACATGACCTGAAGTTTCCTCTGAGGCCAGTC -5'

+843/+867
5'-CCTTTAATGGCCCTAAAACTCATTC-3'
3'-GAATGAGTTTTAGGGCCATTAAAGG-5'

+812/+867
5'-GACTGGCCTCAGAGGAAACTTCAGGTCATGTCCTTTAATGGCCCTAAAACTCATTC-3'
3'-GAATGAGTTTTAGGGCCATTAAAGGACATGACCTGAAGTTTCCTCTGAGGCCAGTC-5'

+812/+867 mutant
5'-GACTGGCC**AA**AGAGGAAACTTCAGG**AA**ATGTCCTTTAATGGCCCTAAAACTCATTC-3'
3'-GAATGAGTTTTAGGGCCATTAAAGGACAT**TT**CCTGAAGTTTCCTCT**TT**GGCCAGTC-5'

RARβ2 COUP-TFII RE (DR8)

5'-GGTGATGTCAGACTAGTTGGGTCATTTG-3'
5'-CAAATGACCCAACTAGTCTGACATCACC-3'

B. Primers used for Chromatin Immunoprecipitation qPCR

Human γ-globin (HBG2) promoter
Fw 5'-CCTTGCCTTGACCAATAGCCTTGACAAGG-3'
Rev 5'-CACAAGCCTGTGGGGCAAGGTGA-3'

Bgl3
Fw 5'-AGTGTTGGGGGAGAAGTGTG-3'
Rev 5'-AATGACCTAATGCCCAGCAC-3'

Globin LCR DNAse I hypersensitive site HS2
Fw 5'- CTGTGTAACCTTCTAAGC -3'
Rev 5'- CAGATAGGAGTCATCACT -3'

Globin LCR DNAse I hypersensitive site HS3
Fw 5'-ATAGACCATGAGTAGAGGGCAGAC-3'
Rev 5'-TGATCCTGAAAACATAGGAGTCAA-3'

Globin Locus 3' HS1
Fw 5'-TCTTCAGCCATCCCAAGACT-3'
Rev 5'-TGGTCTTTTCTGGACACCAC-3'

C. Primers used for Reverse Transcriptase qPCR

Bgl3
Fw 5'-GGGAACAGCCACAAACAAGAAAGA-3'
Rev 5'-AGCCAACCAGGAATTCAGCAGTT-3

γ-globin HBG1/2
Fw 5'-CCATAAAGCACCTGGATGATC-3',
Rev 5'-ATCTGGAGGACAGGGCACTG-3.

FIGURE S4

COUP-TFII binding to RARβ2 promoter

A) EMSA assays showing binding of COUP-TFII to the COUP-TFII response element located within the RAR2 proximal promoter (Left panel; See Fig. S3A for probe sequence). Note that little or no change in mobility is observed by addition of BCL11A-XL protein (lanes 3&4). COUP-TFII can also shift a larger (300bp) RARβ2 promoter fragment (right panel).

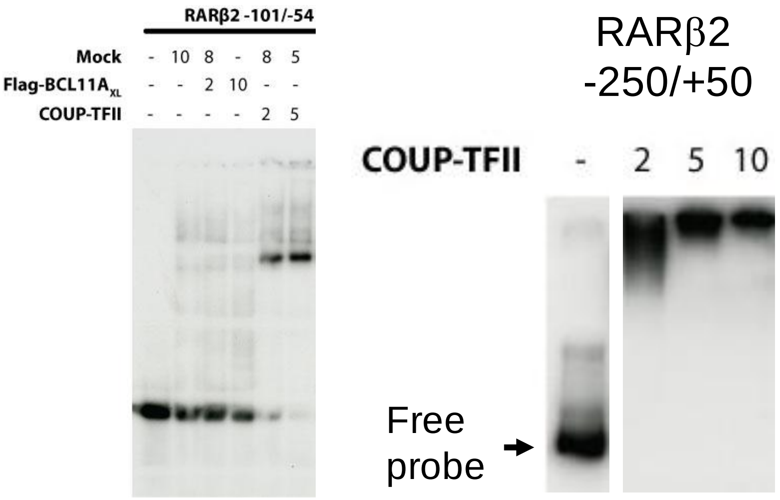

B) Expression of recombinant proteins for use in EMSAs

Western blot detection of BCL11A-XL, COUP-TFII and TLX proteins using the antibodies indicated as described in the Methods.

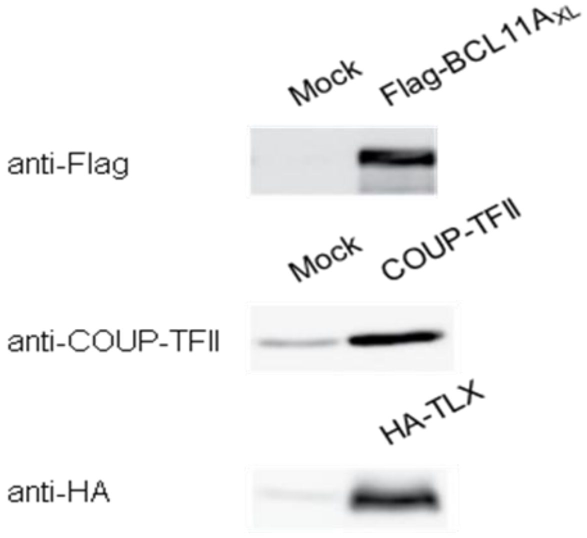

FIGURE S5

A. Detection of BCL11A-XL proteins in leukaemia cell lines.

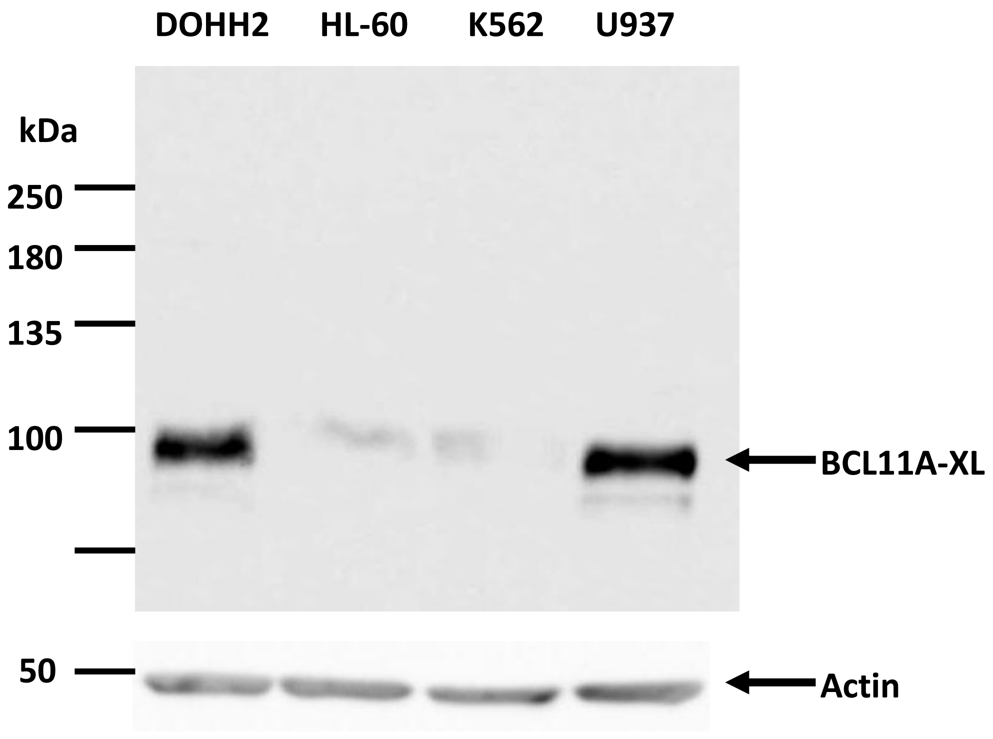

Western blots showing expression levels of BCL11A-XL and β-Actin controls in cell-free extracts of the indicated cell lines. Extracts were prepared as described in Materials & Methods, and approximately 50μg of extract was loaded per lane and resolved on 8% SDS-PAGE gel. The blot was incubated with Anti-BCL11A primary antibody for 3 hours at room temperature, followed by washing and incubation with secondary antibody as described in Methods. The lower portion of the blot was incubated with anti-Actin antibody, as a loading control.

FIGURE S6

A. Direct interaction of BCL11A-XL with Bgl3 sequences

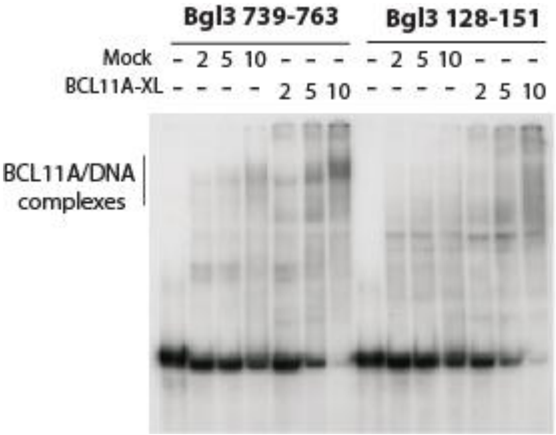

EMSAs were performed as described for Figure 2E to assess direct interaction of BCL11A with Bgl3 sequences. Both probes showed concentration-dependent mobility shifts, however the protein/DNA complexes were diffuse, indicating multivalent interactions, low sequence specificity or complex instability. In contrast BCL11A proteins did not appear to associate directly with the COUP-TFII binding sequence present in the RARb2 (See Fig. S4D).

B. Assessment of COUP-TFII / BCL11A binding to Bgl3 DNA probes.

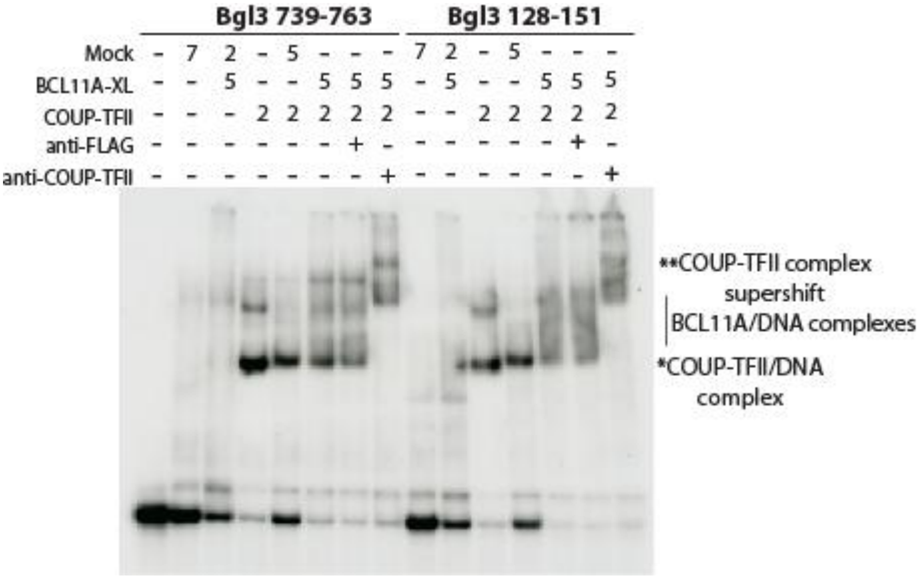

EMSAs and antibody supershift assays were performed as described for Figure 2F. Although addition of FLAG-BCL11A-XL containing extract (but not control) resulted in the emergence of higher molecular weight bands COUP-TFII (compare lanes 4-6; 11-13), we did not observe a strong supershift with α-FLAG antibody (lane 7,14), in contrast to the α-COUPTFII (lane 8,15).

FIGURE S7

(A) RID1 / RID2-Related Motifs in NR-Associated Proteins

					Reported NR Interactions
			FSXXLXXL		
CREBZF	154	AEMQR	FSDLLQRL	LNGIGGCS	NR0;NR1I3;NR2A1;NR3B/C
ARIP4	738	DKILV	FSQSLSTL	ALIEEFLG	NR3C4; (Atrophins)
MPPED2	94	SEVKK	FNDWLGNL	PYEYLIVI	NR2F1;NR2F6
EEF1D	113	LDKSL	FVEWSQAL	LLAPEGSR	NR2F1;NR2F6
RASD1	60	FHRKF	YSIRGEVY	QLDILDTS	NR2F6
SREBP1	809	DGDKE	FSDALGYL	QLLNSCSD	NR0;NR3C4;NR1A1
SREBP2	834	EESCE	FSSALEYL	KLLHSFVD	NR1A1
CEBP/B	75	ERAID	FSPYLEPL	GAPQAPAP	NR1B/C/F;NR3C1
RUNX1	194	PGSLS	FSERLSEL	EQLRRTAM	NR1B/I;NR3C4
RUNX2	228	SKPSL	FSDRLSDL	GRIPHPSM	NR0B2;NR3C4
ASCC3	550	EMTDY	FSRRLEPL	GIIVKELT	NR1A1
BTG1	40	RQLQT	FSQSLQEL	LAEHYKHH	NR1A1;NR1B1
MYST1	330	KFLIA	FSYELSKL	SKLESTVG	NR3C4
MED1	145	KNFDE	FSKHLGKL	VNLYNLPG	NR1;NR2AB;NR3AC;NR4A
BCL2	104	QAGDD	FSRRYRRD	FAEMSSQL	NR4A1
CHD7	12	EDGNI	FSEGLEGL	GECGYPEN	
CHD5	159	TNYKA	FSQFLRPL	IAKKNPKI	
ASB1	184	DVQPR	FSRRLTSL	VVCPLYIS	
ATRX	2035	DKVLV	FSQSLISL	DLIEDFLE	Atrophins
BCORL1	1625	CNWFL	FSDVLKRL	KLSSRIFQ	
BTBD7	190	IDMPM	FSALLHYL	YTGEFGME	

(B) RID1 / RID2-Related Motifs in NRs

NR1F3/RORγ	398	SSIFD	FSHSLSAL	HFSEDEIAL
NR1D2/Reverbβ	484	NSMFE	FSEKLNAL	QLSDEEMSL
NR1D1/Reverbα	518	SAMFD	FSEKLNSL	ALTEEELGL
NR4A1/NUR77	489	DSILA	FSRSLHSL	LVDVPAFAC
NR4A3/NOR1	526	DSIKD	FSLNLQSL	NLDIQALAC
NR5A1/SF1	372	KFIIL	FSLDLKFL	NNHILVKDA
NR5A2/LRH1	452	KFLVL	FSLDVKNL	ENFQLVEGV
NR0B2/SHP	170	KGTIL	FNPDVPGL	QASSHIGHL
NR2F2/COUPTFII	316	KAIVL	FTSDACGL	SDVAHVESL
NR2F1/COUPTFI	323	KAIVL	FTSDACGL	SDACGLSDA
NR2F6/EAR-2	293	KAIAL	FTPDACGL	SDPAHVESL
NR2C2/TR4	515	KAIVL	FSPDHPGL	TSTSQIEKF
NR2C1/TR2	503	KAIVL	FSPDHPSL	ENMEQIEKF
NR2B1/RXRA	371	RAIVL	FNPDSKGL	SNPAEVEAL
NR2A1/HNF4A	290	KAIIF	FDPAKGLS	DPGKIKRLR
NR2E3/PNR	324	KALVL	FKPETRGL	KDPEHVEAL
NR2E1/TLL	289	KCIVT	FKAVPTHS	GSELRSFRN
NR1I2/PXR	307	QAISL	FSPDRPGV	LQHRVVDQL
NR1F1/RORα	456	DEIAL	FSAFVLMS	ADRSWLQEK
NR6A1/GCNF	393	KAINF	LNQDIRGL	TSASQLEQL

(A) Sequence alignments showing the occurrence of RID1/ RID2-related sequences in known NR-binding proteins or other proteins known to function in gene regulation. These sequences were identified using the Prosite Scan tool (www.expasy.org). Interactions with

NR subtypes as recorded in NCBI Gene or protein interaction databases (e.g. BioGRID) are indicated. Reported interactions with atrophins are also indicated. Notably, the Androgen Receptor Interacting Protein ARIP4 (also termed RAD54L2) is a component of the SNF2 family that is reported to function as a cofactor for AR (61). Like TLX, ARIP4 has been reported to interact with both Atrophin (62) and LSD1/CoREST complexes. The leucine zipper protein CREBZF (also known as SMILE or CREB/ATF) was reported to function as a corepressor for a number of NRs including NR3B3/ERRγ, NR1I3/CAR and NR2A1/HNF4α (63,64). The region required to bind NRs in GST pulldown assays was mapped to the sequence 113-202 (63) which comprises a sequence resembling RID1, although it is not yet known if this protein binds NR2E/Fs. A number of other NR2E/F- interacting proteins reported in the literature such as the prospero-related protein PROX1 (65), MPPED1/2 (66), and eEF1D (66), harbour sequences that show weaker resemblance to the RID1, RID2 or ATRO motifs. It remains to be determined whether these sequences are involved in interactions with NR2E/s or other NRs. (B) Sequence alignments showing the occurrence of FSXXLXXL-related sequences in NRs.

Supplementary references

62. Rouleau, N., Domans'kyi, A., Reeben, M., Moilanen, A.M., Havas, K., Kang, Z., Owen-Hughes, T., Palvimo, J.J. and Janne, O.A. (2002) Novel ATPase of SNF2-like protein family interacts with androgen receptor and modulates androgen-dependent transcription. *Molecular biology of the cell*, **13**, 2106-2119.
63. Lim, J., Hao, T., Shaw, C., Patel, A.J., Szabo, G., Rual, J.F., Fisk, C.J., Li, N., Smolyar, A., Hill, D.E. *et al.* (2006) A protein-protein interaction network for human inherited ataxias and disorders of Purkinje cell degeneration. *Cell*, **125**, 801-814.
64. Xie, Y.B., Nedumaran, B. and Choi, H.S. (2009) Molecular characterization of SMILE as a novel corepressor of nuclear receptors. *Nucleic acids research*, **37**, 4100-4115.
65. Xie, Y.B., Park, J.H., Kim, D.K., Hwang, J.H., Oh, S., Park, S.B., Shong, M., Lee, I.K. and Choi, H.S. (2009) Transcriptional corepressor SMILE recruits SIRT1 to inhibit nuclear receptor estrogen receptor-related receptor gamma transactivation. *The Journal of biological chemistry*, **284**, 28762-28774.
66. Song, K.H., Li, T. and Chiang, J.Y. (2006) A Prospero-related homeodomain protein is a novel co-regulator of hepatocyte nuclear factor 4alpha that regulates the cholesterol 7alpha-hydroxylase gene. *The Journal of biological chemistry*, **281**, 10081-10088.
67. Albers, M., Kranz, H., Kober, I., Kaiser, C., Klink, M., Suckow, J., Kern, R. and Koegl, M. (2005) Automated yeast two-hybrid screening for nuclear receptor-interacting proteins. *Molecular & cellular proteomics : MCP*, **4**, 205-213.
